# Ex vivo immune profiling in patient blood enables quantification of innate immune effector functions

**DOI:** 10.1038/s41598-021-91362-5

**Published:** 2021-06-08

**Authors:** Teresa Lehnert, Ines Leonhardt, Sandra Timme, Daniel Thomas-Rüddel, Frank Bloos, Christoph Sponholz, Oliver Kurzai, Marc Thilo Figge, Kerstin Hünniger

**Affiliations:** 1 Research Group Applied Systems Biology, Leibniz Institute for Natural Product Research and Infection Biology ‐ Hans Knöll Institute, Jena, Germany; 2 Center for Sepsis Control and Care (CSCC), Jena University Hospital, Jena, Germany; 3 Research Group Fungal Septomics, Leibniz Institute for Natural Product Research and Infection Biology ‐ Hans Knöll Institute, Jena, Germany; 4 Department of Anesthesiology and Intensive Care Medicine, Jena University Hospital, Jena, Germany; 5 Institute for Hygiene and Microbiology, University of Würzburg, Würzburg, Germany; 6 Institute of Microbiology, Faculty of Biological Sciences, Friedrich Schiller University Jena, Jena, Germany

**Keywords:** Computational models, Infection, Inflammation, Innate immunity, Computational biology and bioinformatics, Immunology

## Abstract

The assessment of a patient’s immune function is critical in many clinical situations. In complex clinical immune dysfunction like sepsis, which results from a loss of immune homeostasis due to microbial infection, a plethora of pro- and anti-inflammatory stimuli may occur consecutively or simultaneously. Thus, any immunomodulatory therapy would require in-depth knowledge of an individual patient’s immune status at a given time. Whereas lab-based immune profiling often relies solely on quantification of cell numbers, we used an ex vivo whole-blood infection model in combination with biomathematical modeling to quantify functional parameters of innate immune cells in blood from patients undergoing cardiac surgery. These patients experience a well-characterized inflammatory insult, which results in mitigation of the pathogen-specific response patterns towards *Staphylococcus aureus* and *Candida albicans* that are characteristic of healthy people and our patients at baseline. This not only interferes with the elimination of these pathogens from blood, but also selectively augments the escape of *C. albicans* from phagocytosis. In summary, our model could serve as a valuable functional immune assay for recording and evaluating innate responses to infection.

## Introduction

Critical illness may be associated with significant alterations in immune function, including both hypo- and hyper-inflammatory states. While impairment of immunity significantly increases the risk of infection, systemic inflammation promotes the development of multiple organ dysfunction syndrome. Therefore, assessment of the immune function of patients is desirable in clinical practice. However, most laboratory tests rely solely on quantification of immune cell populations. For example, the risk of infection during neutropenia is assessed by determining the number of neutrophils found in peripheral blood^1^. Similarly, the CD4^+^ T-cell count is used to quantify the degree of immunosuppression in HIV infection^2^. Although these assays are highly useful in clinical routine, they use quantitative thresholds and do not provide any information about immune cell function^3^. To improve understanding of a patient’s immune status, functional immune monitoring has been attempted using quantification of released proteins as indirect markers, such as IL-6, IL-8, procalcitonin and C-reactive protein^4^. All of these are derived from different immune cells, are mostly pleiotropic and may antagonize each other, all of which limits their clinical use. Sepsis-induced immunosuppression can be measured by reduced expression of the major histocompatibility (MHC) class II molecule HLA-DR on monocytes, which is associated with a diminished antigen-presenting capacity and a shift from pro- to anti-inflammatory cytokine production (reviewed in^5,6^). Similarily, neutrophil surface expression of high-affinity Fcγ receptor I (CD64) has been shown to increase in patients during the early immune response to bacterial infection and in systemic inflammatory response syndrome^7–9^. Several studies have demonstrated that CD64 measured as an index may be useful for the detection and management of sepsis and bacterial infection in neonatal intensive care units and in adult hospital patients^10,11^. However, while surface markers reflect the activation status of immune cells, they do not provide a direct functional readout. Therefore, in a more complex setting, ex vivo stimulation of whole blood with LPS and quantification of cellular cytokine release has been used to quantify immune function. LPS-induced TNF-α release by monocytes in whole blood tends to be less in immunosuppressed patients compared to healthy individuals^12,13^. Nevertheless, this approach relies on a secreted cytokine as an indirect marker of cellular immune function. The most functional read-out described so far is the assessment of neutrophil dysfunction in critically ill patients by measuring neutrophil capacity to clear zymosan particles ex vivo^3,14^. However, these assays selectively involve neutrophils and require prior isolation of the cells, which may alter their function, especially in patients with activated neutrophils^15^.

In previous studies, we applied a systems biology approach to investigate the immune response to pathogens in blood from healthy individuals^16–18^. Using ex vivo whole-blood infection in combination with biomathematical modeling enabled the calculation of functional parameters for blood immune cells, such as kinetic rates for cell migration and pathogen uptake. Based on these results, we have performed a pilot study to investigate whether a systems biology approach allows quantification of immune function in a clinical setting. We selected patients undergoing cardiac surgery with extracorporeal circulation. Such patients receive a standardized anesthesia regimen and operation procedure and have a well-characterized inflammatory response (e.g. increase of blood lipopolysaccharide and β-D-glucan levels), which enables analysis of immune function before and after the insult^19–21^. By determining functional parameters of innate immune cell populations after ex vivo whole-blood bacterial (*Staphylococcus aureus*) and fungal (*Candida albicans*) infection, we show that post-surgery inflammation results in attenuation of inter-patient and inter-pathogen differences in immune response patterns. Moreover, our model revealed enhanced immune escape by *C. albicans* that was not observed for *S. aureus*, indicating pathogen-specific adaptation to an altered immune status.

## Results

### Pathogen-specific immune response patterns during bacterial and fungal whole-blood infection

**Figure 1:**
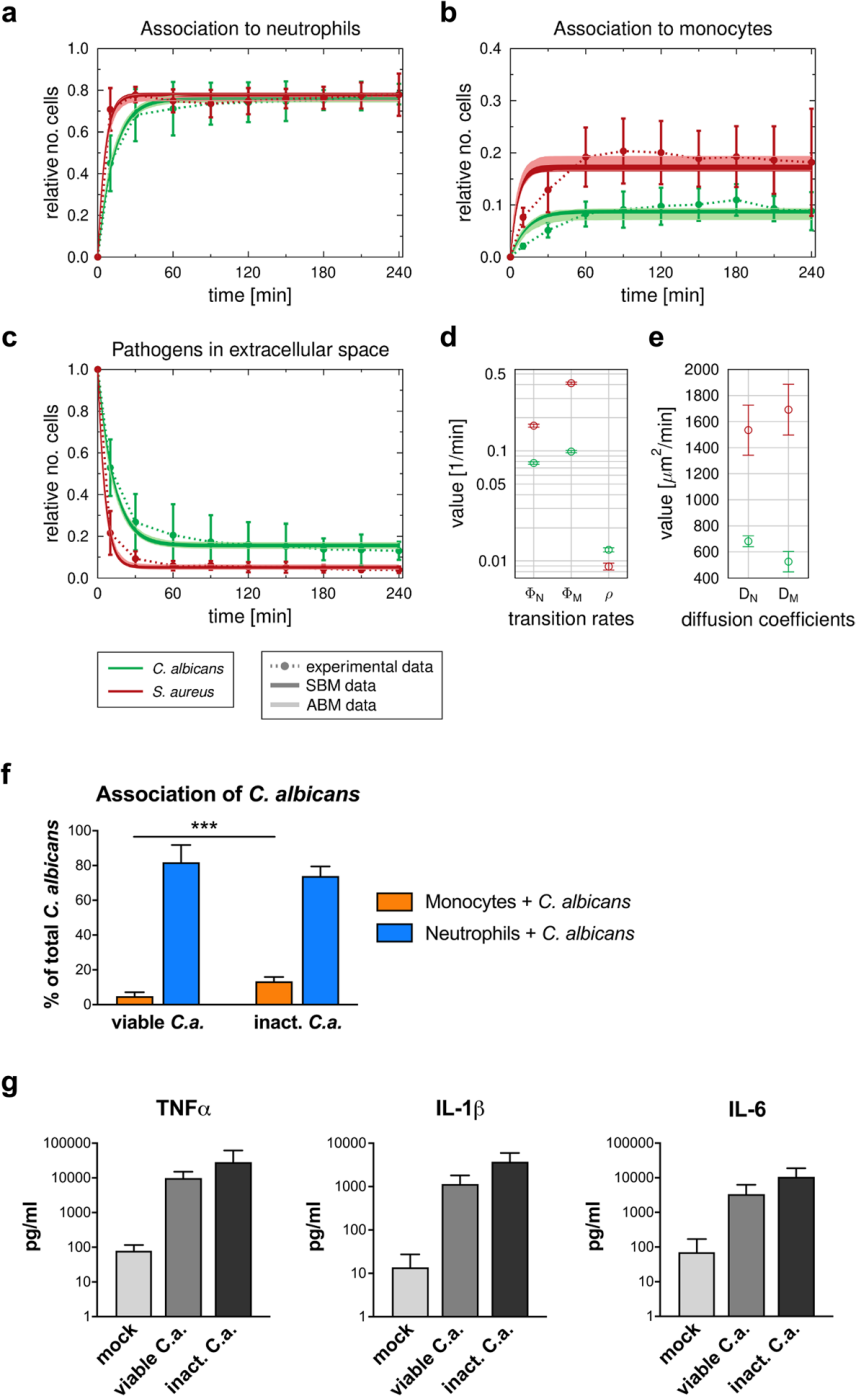
Comparison of the dynamics of host–pathogen interaction during *C. albicans* and *S. aureus* infection in healthy blood. (**a**–**c**) Results of fitting the state-based model (SBM) and the agent-based model (ABM) to experimental data. Simulated dynamics of the combined units (solid line) were obtained by fitting the SBM (dark color) and the ABM (light color) to the experimentally measured association kinetics (dotted line). Experimental data were gained from whole-blood infection assays with either *C. albicans* (green, n = 10) or *S. aureus* (red, n = 7). SBM: line thickness represents SD obtained by 50 simulations with transition rate values sampled within their corresponding SD. ABM: line thickness represents the standard deviations obtained from 30 stochastic simulations with the estimated diffusion coefficients. (**a**) and (**b**), dynamics of the combined units *P*_*N*_ and *P*_*M*_, which correspond to the experimental data on pathogens associated with neutrophils and monocytes. (**c**) kinetics of the combined unit *P*_*E*_ together with experimentally measured kinetics of either fungal or bacterial cells in extracellular space. (**d**) Mean values (± SD) of transition rate values obtained by fitting the SBM to experimental data using simulated annealing. The rate of phagocytosis by neutrophils (${\phi }_{N}$) and by monocytes (${\phi }_{M}$) as well as the rate for immune escape ($\rho$) are depicted for infection scenarios with either *C. albicans* (green circle) or *S. aureus* (red circle). (e) Diffusion coefficients for neutrophils $({D}_{N})$ and monocytes $({D}_{M})$ were estimated by fitting the ABM to the experimental data for *C. albicans* (green circle) and *S. aureus* (red circle), respectively. Mean and SD are calculated from all parameter sets with a mean LSE within the SD of the optimal parameter set. (**f**) Association of viable and inactivated *C. albicans* with blood monocytes and neutrophils after 60 min quantified using flow cytometry. Significance was estimated using the unpaired, two-sided Student *t* test (***P < 0.001). (**g**) Release of monocyte-derived cytokines (TNF-α, IL-1β, IL-6) in plasma samples generated from 4 h whole-blood infection experiments in response to viable and inactivated *C. albicans* cells was investigated. Bars are shown as means ± SD of at least 3 independent experiments with whole blood from different donors.

Prior analyses in whole blood have been carried out using viable pathogens^16^. However, quantification of immune cell function in patient blood samples requires the use of inactivated stimuli to avoid the effects of pathogen inactivation by antibiotics in patient blood. Thus, we quantified immune cell functional parameters for two inactivated pathogens, *Staphylococcus aureus* (bacterial) and *Candida albicans* (fungal). During a 4-h time course, neutrophils were the predominant immune cell type to interact with both pathogens (Fig. 1a). Both pathogens showed a similar association with neutrophils after 60 min of blood infection. However, the early 10 min time point revealed much faster association of *S.* *aureus* with neutrophils and monocytes (70.8 ± 10.2% and 7.6 ± 1.8%, respectively) compared with *C.* *albicans* (45.0 ± 13.3% and 3.1 ± 0.5%, respectively) (Fig. 1a,b). Consistent with the different association kinetics, *C.* *albicans* was cleared more slowly than *S.* *aureus* (10 min *p.i.:* extracellular *C.* *albicans* 52.9 ± 13.6%, extracellular *S.* *aureus* 21.6 ± 10.5%, Fig. 1c). Notably, 4 h after infection, a larger population of *C. albicans* cells remained extracellular compared to *S.* *aureus* (extracellular *C.* *albicans* 13.0 ± 4.3%, extracellular *S.* *aureus* 4.0 ± 1.5%). Comparison of these data with previous studies and a new set of experiments using viable *C.* *albicans* showed similar patterns overall, but also revealed a significantly higher number of inactivated fungal cells associated with monocytes (60 min *p.i.*: viable *C.* *albicans* 4.9 ± 2.3%, inactivated *C.* *albicans* 13.4 ± 2.5%, *P* < 0.001) (Fig. 1f and^16^). Association with lymphocytes could not be detected for either pathogen regardless of their viability. Whole-blood infection with *C. albicans* in either an active or inactive state induced a strong and comparable secretion of monocyte-derived cytokines (TNF-α, IL-1β and IL-6), whereas only low cytokine levels could be detected in mock-infected blood (Fig. 1g).

In order to comparatively quantify the functional properties of immune cells we calibrated the state-based virtual infection model (SBM) to the experimental data (see “Methods” section, Table 1, Fig. 1d). The coefficients of variation of the resulting transition rates are very small (< 7%) and the corresponding dynamics of the three combined units are similar to the mean values of the respective experimental data, indicating a good match to the experimental data (see Fig. 1a–c). By comparing the resulting transition rate values, we observed significantly higher phagocytosis rates for *S. aureus* than for *C. albicans* (for neutrophils ${\phi }_{N}^{S.a.}/{\phi }_{N}^{C.a.} =2.2$ with $P{^{\prime}}<0.001$, for monocytes ${\phi }_{M}^{S.a.}/{\phi }_{M}^{C.a.}=4.2$ with $P{^{\prime}}<0.001$). The opposite ratio was found with respect to the rate of immune escape ($\rho )$, (${\rho }^{S.a.}/{\rho }^{C.a.} =0.7$ with $P{^{\prime}}<0.001$). These parameter differences lead to an overall faster removal of *S. aureus* cells from the extracellular space and a higher amount of immune-evading *C. albicans* at 240 min after infection (Fig. 1c).

**Table 1:** Transition rate values of the SBM for *C. albicans* and *S. aureus* infection of blood samples taken from either healthy volunteers or HLM patients before cardiac surgery (pre-operative), immediately after surgery (post-operative) and one day after admission to intensive care (post-operative + 1d).

Transition rate	Mean $\times {10}^{-2}\left[{\mathrm{min}}^{-1}\right]\pm$ SD $\times {10}^{-2}[{\mathrm{min}}^{-1}]$ (CV $[\%]$)							
	Healthy		Pre-operative		Post-operative		Post-operative + 1d	
	*C. albicans*	*S. aureus*	*C. albicans*	*S. aureus*	*C. albicans*	*S. aureus*	*C. albicans*	*S. aureus*
${\phi }_{N}$	7.75 ± 0.20 (2.6)	16.94 ± 0.55 (3.3)	4.45 ± 0.14 (3.1)	10.65 ± 0.36 (3.3)	16.67 ± 0.36 (2.2)	20.86 ± 0.54 (2.6)	23.24 ± 0.77 (3.3)	25.20 ± 0.72 (2.9)
${\phi }_{M}$	9.87 ± 0.23 (2.4)	41.19 ± 9.45 (2.3)	9.29 ± 0.28 (3.0)	24.74 ± 0.64 (3.3)	94.41 ± 1.98 (2.1)	97.94 ± 0.98 (3.3)	72.28 ± 2.23 (3.1)	84.60 ± 2.24 (3.3)
$\rho$	1.26 ± 0.06 (4.4)	0.89 ± 0.06 (7.0)	1.32 ± 0.05 (3.8)	1.26 ± 0.07 (5.3)	1.88 ± 0.08 (4.3)	0.90 ± 0.05 (5.4)	2.86 ± 0.10 (3.5)	1.43 ± 0.10 (7.0)

The transition rate values are given by the phagocytosis rate ${\phi }_{N}$ of neutrophils, the phagocytosis rate ${\phi }_{M}$ of monocytes and the rate for acquiring immune escape $\rho$.

Based on the estimated parameters of the SBM, we also simulated the infection scenario with both pathogens in an agent-based model (ABM) (see “Methods” section). Similar to the SBM dynamics, the resulting dynamics of the ABM are in agreement with the experimental data (see Fig. 1a–c). Diffusion coefficients for neutrophils and monocytes are two to four times higher for *S. aureus* infection $({D}_{N},{D}_{M} )=(1500 \mu {\mathrm{m}}^{2}/\mathrm{min}, 1800 \mu {\mathrm{m}}^{2}/\mathrm{min})$ compared to *C. albicans* $\left({D}_{N},{D}_{M}\right)=(700 \mu {\mathrm{m}}^{2}/\mathrm{min}, 450 \mu {\mathrm{m}}^{2}/\mathrm{min})$. Furthermore, the diffusion coefficient for neutrophils is higher than for monocytes during *C. albicans* infection and vice versa for *S. aureus* infection (Fig. 1e).

### Cardiac surgery results in neutrophil mobilization and immune activation

**Figure 2:**
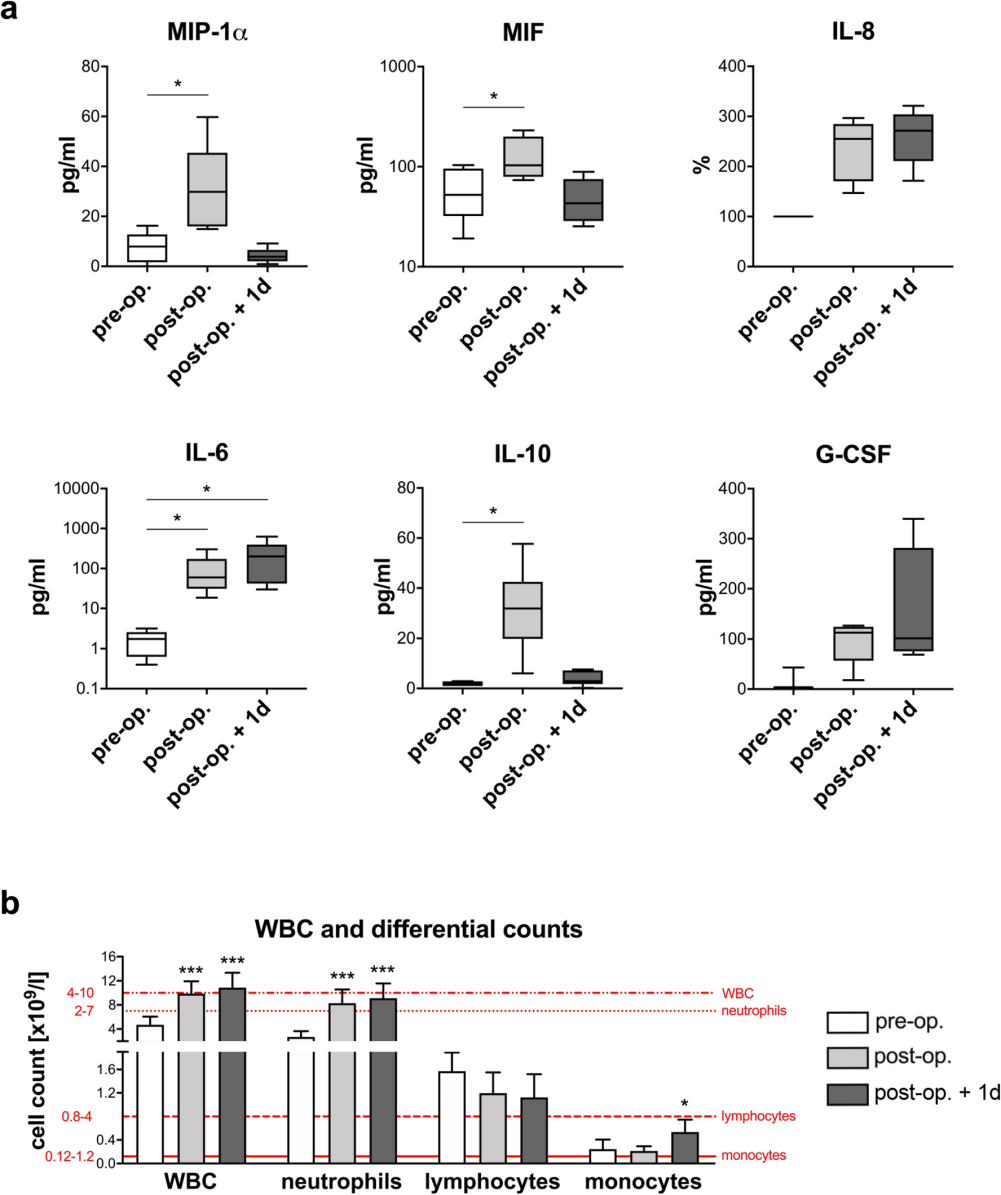
Blood after surgery shows changes in cytokine profiles and peripheral differential cell counts. Blood samples from six HLM patients taken before cardiac surgery (pre-operative), immediately after surgery (post-operative) and one day after admission to intensive care (post-operative + 1d) were analyzed for (**a**) cytokine levels (MIP-1α, MIF, IL-8, IL-6, IL-10, G-CSF) using Luminex technology and (**b**) white blood cell (WBC) as well as neutrophil, lymphocyte and monocyte counts using an automated hematology analyzer. Reference ranges of leukocytes are indicated in red. The plasma concentrations of cytokines are presented in pg/ml, except for IL-8. Levels for IL-8 in post-operative and post-operative + 1d blood samples were normalized to respective plasma levels within pre-operative blood (set to 100%) for each donor and means ± SD of the calculated percentages are shown. Significance was estimated using the unpaired, two-sided Student *t* test and shown as *P < 0.05, ***P < 0.001.

**Figure 3:**
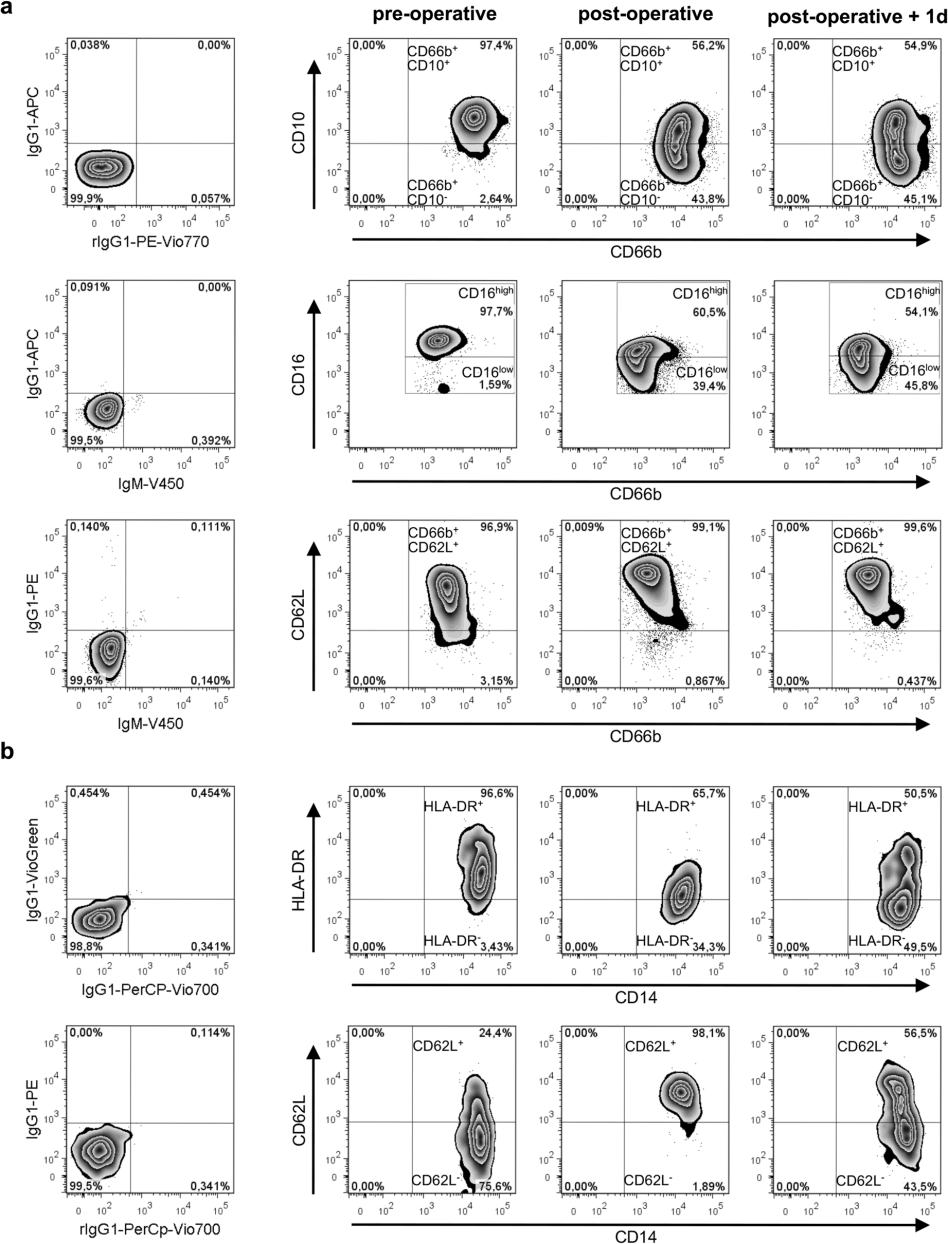
Surface phenotypes of monocytes and neutrophils after surgery indicate an increased number of immature cells in blood of HLM patients. Flow cytometry analysis of CD66b^+^ neutrophils (**a**) and CD14^+^ monocytes (**b**) from whole blood of HLM patients taken before cardiac surgery (pre-operative), immediately after surgery (post-operative) and one day after admission to intensive care (post-operative + 1d) are shown. (**a**) Immature phenotype of CD66b^+^ blood neutrophils was analyzed by surface expression of CD10, CD16 and CD62L. Representative zebra plots show a change of the surface phenotype by the shift of the population to the lower right quadrant for CD10 and CD16 and to the higher right quadrant for CD62L. (**b**) Surgery-induced changes on CD14^+^ blood monocytes are represented by zebra plots of HLA-DR and CD62L expression patterns. Left plots show proper gate setting defined by isotype control stainings.

The data generated so far clearly show that a combination of ex vivo blood infection and virtual infection modelling allows quantification of immune cell functions. To address the feasibility of this approach in a clinical setting, we used blood samples from patients undergoing cardiac surgery for mitral valve insufficiency (heart lung machine (HLM) patients). Six patients (4 female, 2 male) aged between 52 and 74 years were recruited. All received minimally invasive mitral valve replacement or reconstruction surgery via lateral thoracotomy on cardiopulmonary bypass (CPB). Anesthesia was induced by Propofol, Sufentanyl and Rocuronium and sustained by Sufentanyl and Sevofluran before and Propofol during CPB. Three patients underwent additional surgery such as tricuspid valve surgery, cryoablation, atrial appendage closure and atrial septal defect closure. Bypass time ranged from 99 to 220 min, the average cumulative fluid balance on the day of surgery was 3316 ± 2486 ml (see Supplementary Table S1 for individual patient data). All patients were extubated on the day of surgery and none had any major complications. The only post-operative infection was a urinary tract infection more than two weeks after surgery. After informed consent, blood samples were taken before cardiac surgery (pre-operative), directly after surgery (post-operative) and one day after admission to intensive care (post-operative + 1d). These defined time points together with the low variance in surgical time and duration of extracorporeal circulation as well as the time-defined, strong and homogeneous inflammatory stimulus allowed us to clearly distinguish between inter-individual differences and the effects of inflammation. Levels of the pro-inflammatory cytokines IL-6, IL-8, MIP-1α and MIF and the anti-inflammatory cytokine IL-10 were increased in post-operative blood (Fig. 2a), whereas TNF-α and IL-1β were not detectable in plasma samples before and after surgery. Elevated levels of IL-6 and IL-8 were sustained until one day after surgery. Furthermore, the mean total white blood cell count increased after surgery (pre-operative: 4.70 ± 1.37 × 10^﻿9^/L, post-operative: 9.87 ± 2.05 × 10^9^/L, post-operative + 1d: 10.89 ± 2.45 × 10^9^/L) (Fig. 2b, see Supplementary Table S1 for individual patient data). Quantitative analysis of white blood cells revealed a significant increase in neutrophils (both post-operative and post-operative + 1d) and monocytes (post-operative + 1d), whereas lymphocyte counts remained stable. Neutrophilia following cardiac surgery is mediated by granulocyte colony-stimulating factor (G-CSF)-induced bone marrow neutrophil release^22^. G-CSF was markedly higher in plasma samples obtained directly and one day after surgery compared to pre-operative blood (Fig. 2a). Furthermore, whereas pre-operative blood was characterized by a homogenous neutrophil population that expressed abundant CD10 and high CD16 surface expression (mature neutrophils), the neutrophil population after surgery contained a CD10-negative subpopulation with reduced CD16 and increased L-selectin (CD62L) expression, indicating recruitment of immature neutrophils (Fig. 3a). Post-operatively, the CD10^neg^ neutrophil subpopulation accounted for 49.3 ± 4.1% of the total neutrophil population and remained fairly stable after one day (46.7 ± 2.6%). Whereas no changes in monocyte counts could be observed between the pre- and post-operative time points, total monocyte numbers were significantly increased one day after surgery (pre-operative: 0.24 ± 0.16 × 10^9^/L, post-operative + 1d: 0.54 ± 0.21 × 10^9^/L, *P* < 0.05) (Fig. 2b, see Supplementary Table S1 for individual patient data). Expression of the MHC class II antigen HLA-DR on monocytes was markedly decreased after surgery (Fig. 3b). Together with the increased CD62L expression, this suggests that surgery induced an immature monocyte state.

### Pro-inflammatory stimulus during cardiopulmonary bypass mitigates inter-patient and inter-pathogen differences in immune response patterns

**Figure 4:**
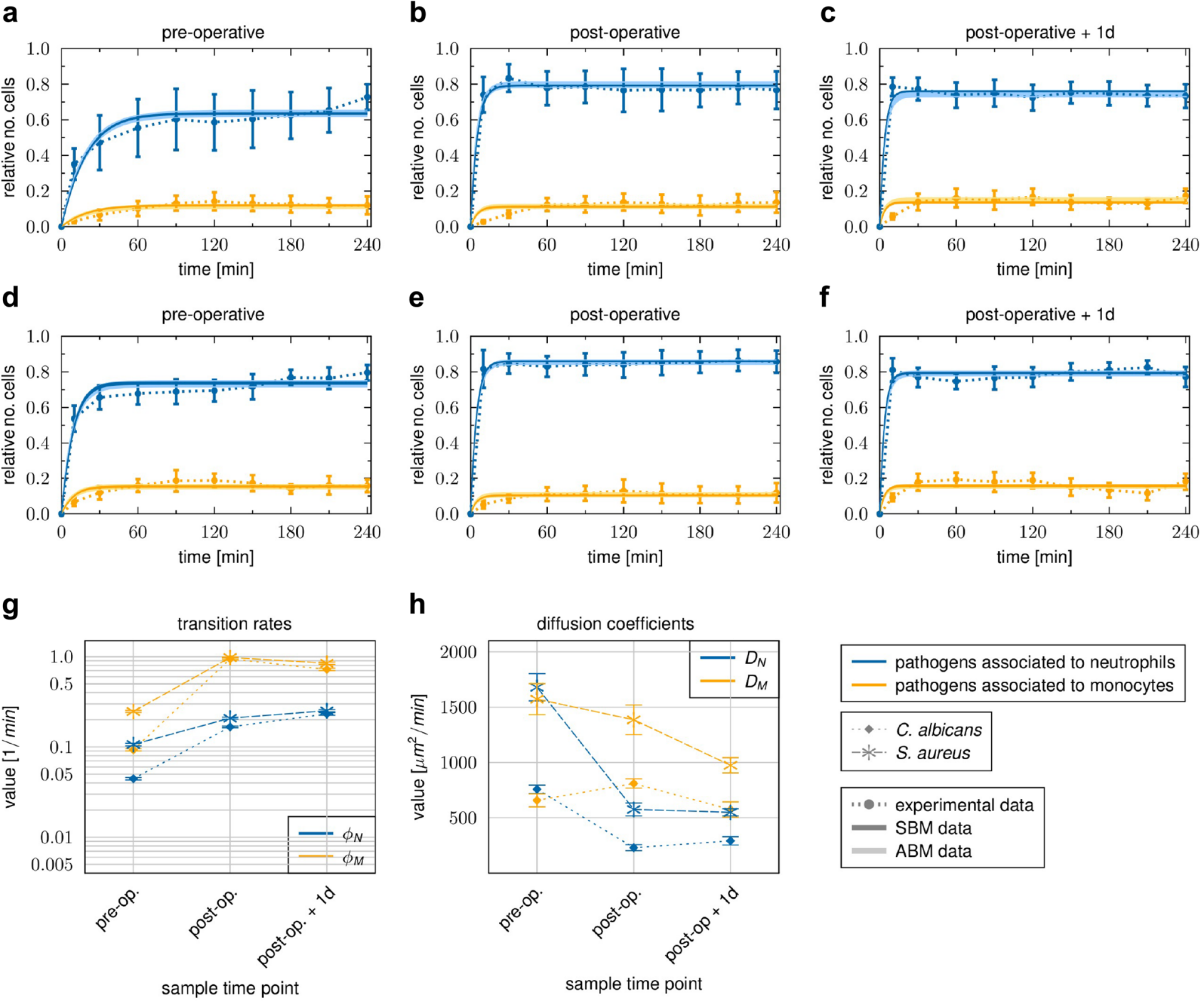
Time courses of pathogen association to immune cells observed in whole-blood samples of HLM patients. Blood samples were taken before cardiac surgery (pre-operative), immediately after surgery (post-operative) and one day after admission to intensive care (post-operative + 1d). Time-resolved experimental data (dotted line) were obtained by whole-blood infection assays with either *C. albicans* (**a**–**c**) or *S. aureus* (**d**–**f**). Data points and error bars refer to the means and SD of blood samples from six HLM patients. The simulated dynamics of the combined units (solid line) were obtained by fitting the state-based model (SBM, dark color) and the agent-based model (ABM, light color) to the experimental data. The thickness of the results from the SBM represents the SD obtained by 50 simulations with transition rate values that were sampled within their corresponding SD. The thickness of the results from the ABM represents the SD obtained from 30 stochastic simulations of the ABM with the estimated diffusion coefficients. (**g**) Transition rate values of the SBM resulting from fitting the model to experimental data of either *C. albicans* or *S. aureus* infection in blood samples from HLM patients. The transition rate values are given for the phagocytosis rate ${\phi }_{N}$ of neutrophils and the phagocytosis rate ${\phi }_{M}$ of monocytes. (**h**) The diffusion coefficients are given for neutrophils ${D}_{N}$ and monocytes ${D}_{M}$. Mean and SD are calculated from all parameter sets with a mean LSE that lies within the SD of the optimal parameter set.

To quantify functional immune parameters in whole blood after cardiopulmonary bypass, we perfomed whole-blood infection with inactivated *S.* *aureus* or *C.* *albicans* as described above. These analyses revealed clear changes in innate immune response patterns against *C. albicans* and *S. aureus*. In blood collected after surgery, association of the pathogens with neutrophils and monocytes was increased in comparison to the pre-operative time point and occurred more quickly (Fig. 4). For instance, 10 min post infection, 35.1 ± 8.8% of *C.* *albicans* and 53.7 ± 7.2% of *S.* *aureus* cells*,* respectively, were associated with neutrophils in pre-operative blood (Fig. 4a,d), compared to 78.5 ± 5.1% for *C.* *albicans* and 81.1 ± 6.7% for *S. aureus* one day after surgery (Fig. 4c,f). To test whether the altered association kinetics could be explained by changes in leukocyte numbers or indicate functional differences, we fitted the SBM to the association kinetics and used the means of the measured immune cell counts as input for the model simulations. These analyses clearly showed that the faster association kinetics after surgery could only be explained by a significant increase in the phagocytosis rates of neutrophils and monocytes and not solely by increased immune cell counts (Fig. 4g). Importantly, pathogen-specific response patterns that were clearly visible in blood from healthy donors and in samples taken before surgery were absent after the pro-inflammatory stimulus. More specifically, *S. aureus* had increased and faster association with neutrophils and monocytes than *C. albicans* in blood taken before surgery (Fig. 4a,d). Comparing the transition rates for phagocytosis by neutrophils (${\phi }_{N})$ and monocytes (${\phi }_{M})$ between both pathogens revealed significantly higher values for *S. aureus* (${\phi }_{N}^{S.a.}/{\phi }_{N}^{C.a.} =2.4$, ${\phi }_{M}^{S.a.}/{\phi }_{M}^{C.a.} =2.7$) (Fig. 4g). In contrast, association kinetics for *C.* *albicans* and *S.* *aureus* as well as the corresponding phagocytosis rates for neutrophils and monocytes were almost similar in blood after surgery (e.g. post-operative + 1d: ${\phi }_{N}^{S.a.}/{\phi }_{N}^{C.a.}=1.1$, ${\phi }_{M}^{S.a.}/{\phi }_{M}^{C.a.}=1.2$; Fig. 4g).

**Figure 5:**
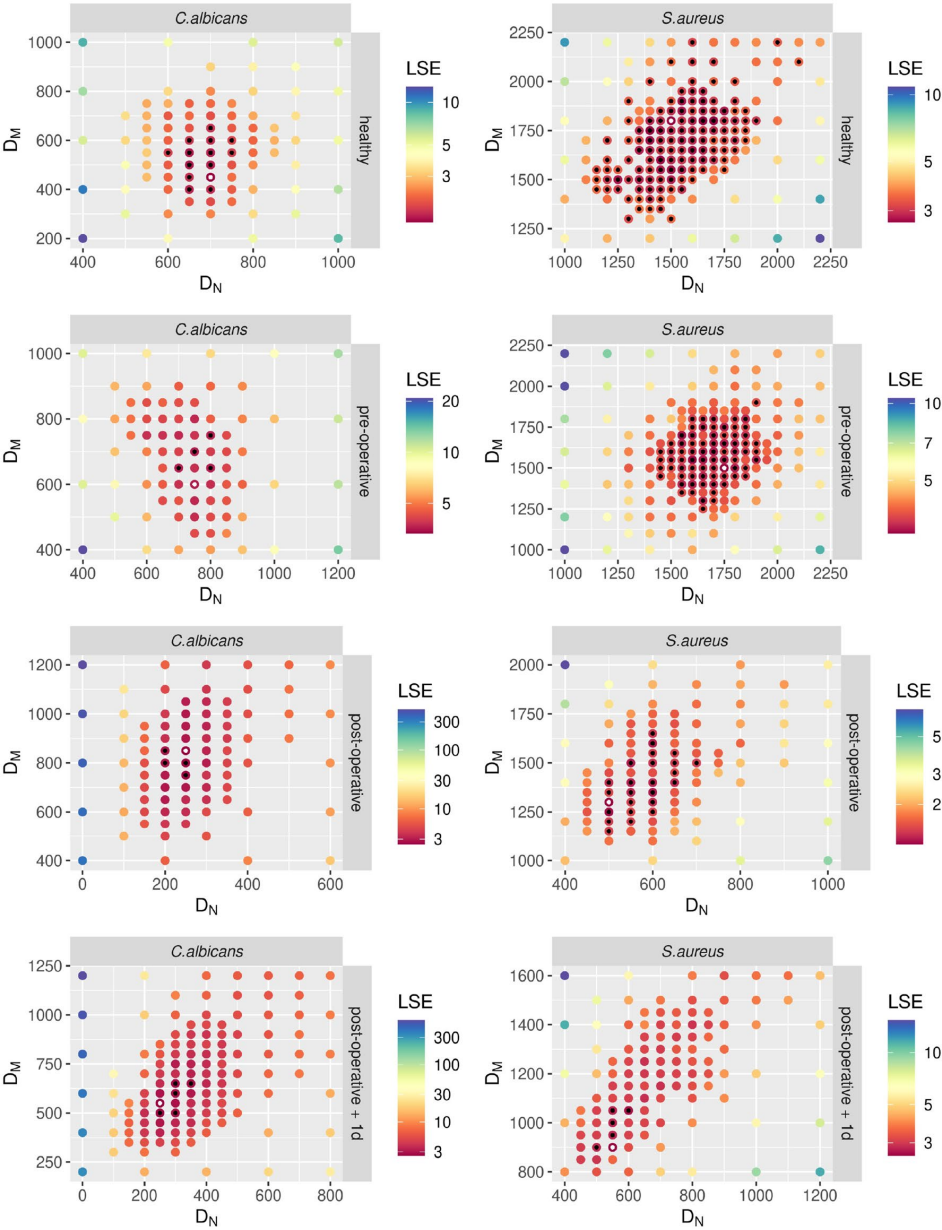
Results of fitting the agent-based model (ABM) to the experimental data from *C. albicans* and *S. aureus* infection using the method of adaptive regular grid search. The parameter space is shown for fitting the ABM to experimental data, where blood samples from healthy donors and HLM patients before surgery (pre-operative), immediately after surgery (post-operative) and one day after admission to intensive care (post-operative + 1d) were infected with either *C. albicans* (left column) or *S. aureus* (right column). Colors of the points refer to the weighted least squares error $E\left(\overrightarrow{p}\right)$ for each parameter set $\overrightarrow{p}=\left({D}_{N},{D}_{M}\right)$. The optimal parameter set is marked with a white dot. All parameter sets with a mean LSE that lies within the SD of the optimal parameter set are marked with a black dot.

In addition, we investigated immune cell migration in blood after surgery compared to the pre-operative time point using the ABM. As in healthy donors, diffusion coefficients for monocytes and neutrophils during *S. aureus* infection exceeded diffusion coefficients during *C. albicans* infection by a factor of two to three (Figs. 4h, 5). Infection of post-operative blood with *C. albicans* resulted in a decrease in ${D}_{N}$ by a factor of ${D}_{N}^{pre-OP }/{D}_{N}^{post-OP }=3.0$ and an increase in ${D}_{M}$ by a factor of ${D}_{M}^{pre-OP }/{D}_{M}^{post-OP }\approx 0.7$ (Figs. 4h, 5). Similarly, ${D}_{N}$ was decreased by a factor of ${D}_{N}^{pre-OP }/{D}_{N}^{post-OP }=3.5$ during *S. aureus* infection. Contrary to confrontation with *C. albicans,* ${D}_{M}$ was not increased in *S. aureus-*infected post-operative blood, but rather slightly decreased (Figs. 4h, 5)*.* Furthermore, ${D}_{N}$ did not change from the post-operative time point to one day after surgery for both species. However, in the case of ${D}_{M}$ we observed a decrease for both species by a factor of approximately ${D}_{M}^{post-OP }/{D}_{M}^{post-OP +1d }=1.5$. Comparing the values for ${D}_{N}$ and ${D}_{M}$ between the pathogens for each sample time point showed that both diffusion constants were higher in the presence of *S. aureus* compared to *C. albicans* for all time points. Similar to the immune cell association and phagocytosis rates, differences in neutrophil and monocyte diffusion between the fungal and bacterial pathogen decreased after surgery (Fig. 4h).

In addition to reducing differences in the responses to the two pathogens, the inflammatory stimulus decreased inter-individual differences in the association of pathogens with immune cells. For instance, the percentage standard deviation of pathogens that interacted with neutrophils decreased from 32 to 10% for *C.* *albicans* (Fig. 4a,c) and 13.5% to 8% for *S.* *aureus* infection (Fig. 4d,f) between samples collected pre-operatively and one day after surgery.

Taken together, the kinetics of immune responses were accelerated after the pro-inflammatory stimulus independent of the microbial trigger. This resulted not only from the large increase in immune cell numbers, but also from increased transition rates (see Fig. 4g). As a consequence, pathogen-specific and inter-individual differences were less pronounced after surgery.

### Monocyte activation by *C. albicans* and *S. aureus* is dampened after cardiopulmonary bypass

**Figure 6:**
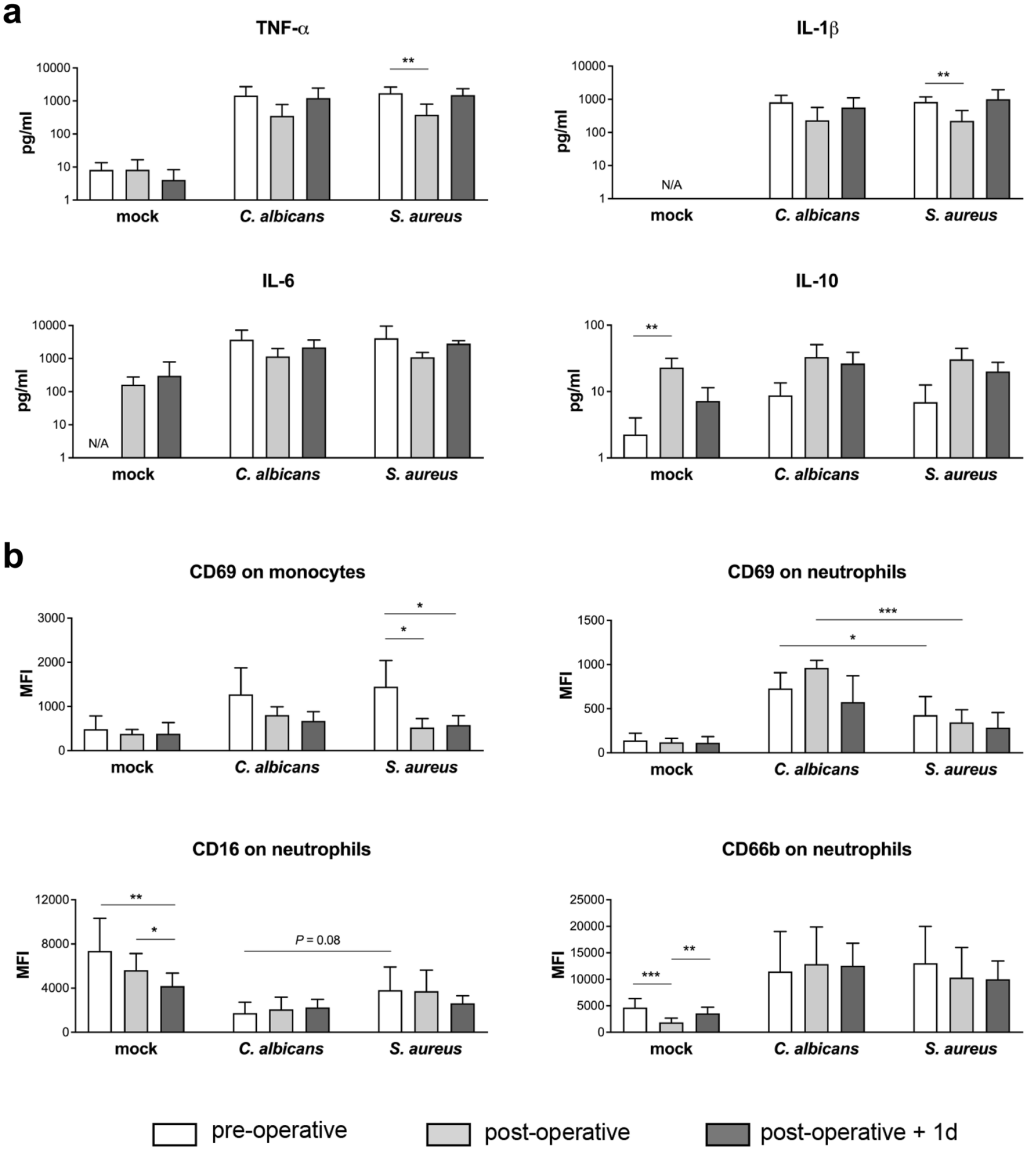
Changes in cytokine secretion and innate immune cell activation in whole blood from HLM patients after surgery. Blood samples from HLM patients were taken before cardiac surgery (pre-operative, non-filled bars), directly after surgery (post-operative, light grey bars) and one day after admission to intensive care (post-operative + 1d, dark grey bars) and either mock-infected, treated with *C. albicans* or *S. aureus* for 4 h. (**a**) Plasma levels of TNF-α, IL-1β, IL-6 and IL-10 were quantified and bars are shown as means ± SD. Results are presented as pg/ml; N/A stands for values not available. (**b**) Surface marker expression was analyzed on the total monocyte population and on pathogen-associated neutrophils by flow cytometry. Data shown are mean fluorescence intensity (MFI) ± SD. Significance is shown as *P < 0.05; **P < 0.01; ***P < 0.001, unpaired, two-sided Student *t* test.

We further investigated consequences of cardiopulmonary bypass surgery-induced inflammation on activation of innate immune cells during infection. Activation of monocytes was analyzed by their surface expression of early activation antigen CD69 and cytokine secretion in response to microbial confrontation, and was reduced after cardiac surgery. The presence of *C.* *albicans* or *S.* *aureus* induced increased release of the monocytic cytokines TNF-α, IL-1β and IL-6 in blood from all three time points tested in HLM patients compared to the corresponding mock-treated samples (Fig. 6a). However, the induced levels were markedly lower in post-operative blood during both *C.* *albicans* and *S.* *aureus* infection (*C. albicans*: TNF-α: pre-operative 1456 ± 1248 pg/ml, post-operative 355 ± 428 pg/ml, *P* = 0.068; *S.* *aureus*: TNF-α: pre-operative 1730 ± 903 pg/ml, post-operative 380 ± 427 pg/ml, *P* < 0.01). The reduced pro-inflammatory cytokine release suggests down-regulation of monocyte activity, which could have been mediated by increased IL-10 levels detected in blood obtained directly after surgery (see Fig. 2a). Neither *C.* *albicans* nor *S.* *aureus* infection was able to induce further IL-10 secretion compared to mock-treated post-operative samples (Fig. 6a). Plasma concentrations of IL-10 almost returned to pre-operative values after one day in the ICU, and accordingly, release of monocytic cytokines was increased again in response to both pathogens (TNF-α: *C.* *albicans* 1229 ± 1234 pg/ml, *S.* *aureus* 1507 ± 852 pg/ml). In line with the cytokine data, elevated CD69 surface levels could be detected on monocytes 4 h after inoculation of *C.* *albicans* (MFI 1276 ± 598) or *S.* *aureus* (MFI 1447 ± 597) compared to mock-infection (MFI 488 ± 298) in pre-operative blood without any quantitative differences between the pathogens (Fig. 6b). A less pronounced response was detected in blood after surgery (*S.* *aureus*: post-operative MFI 524 ± 202, post-operative + 1d MFI 580 ± 212). Together with reduced monocytic cytokine release, these data indicate decreased monocyte stimulation following infection of blood after surgery. Analysis of the surface phenotype of neutrophils revealed significant differences in the expression of CD16 and CD66b between mock-infected control samples in blood taken before and after surgery as well as after one day in the ICU that could be explained by the inflammation-dependent recruitment of immature neutrophils (Fig. 6b). Activation was largely restricted to those neutrophils which had phagocytosed either *C.* *albicans* or *S.* *aureus*, and was stronger in response to *C.* *albicans*, shown by a more pronounced increase in CD69 (pre-operative: mock-infected MFI 141 ± 82, *C.* *albicans*-infected MFI 730 ± 178, *S.* *aureus*-infected MFI 427 ± 211) and down-regulation of CD16 (pre-operative: mock-infected MFI 7359 ± 2962, *C.* *albicans*-infected MFI 1721 ± 998, *S.* *aureus*-infected MFI 3812 ± 2108). However, the decrease in surface CD16 and up-regulation of the degranulation marker CD66b on pathogen-associated neutrophils was equal in blood from all three time points for the respective infections, even though surface levels of both activation markers were different on neutrophils in pre- and post-operative blood. Comparable results were observed for CD69 expression.

### Inflammatory stimulus during cardiopulmonary bypass induces enhanced *C. albicans* immune escape

**Figure 7:**
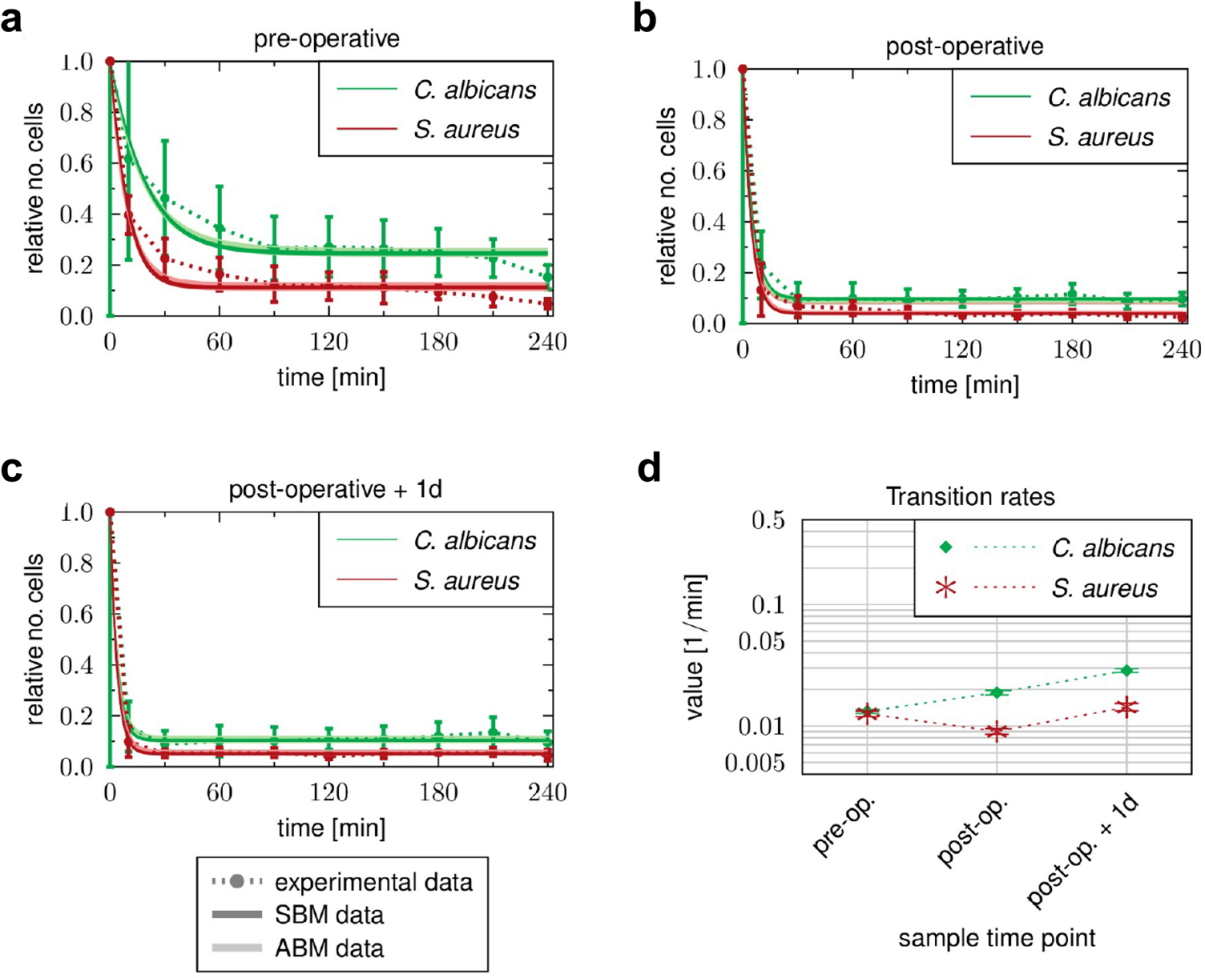
Time courses of extracellular pathogens observed in whole-blood samples of HLM patients. Blood samples were taken (**a**) before cardiac surgery (pre-operative), (**b**) immediately after surgery (post-operative) and (**c**) one day after admission to intensive care (post-operative + 1d). Time-resolved experimental data (dotted line) were obtained by whole-blood infection assays with either *C. albicans* (green) or *S. aureus* (red). Data points and error bars refer to the means and SD of blood samples from six HLM patients. The simulated dynamics of the combined units (solid line) were obtained by fitting the state-based model (SBM, dark color) and the agent-based model (ABM, light color) to the experimental data. The thickness of the results from the SBM represents the SD obtained by 50 simulations with transition rate values that were sampled within their corresponding SD. The thickness of the results from the ABM represents the SD obtained from 30 stochastic simulations of the ABM with the estimated diffusion coefficients. (**d**) Transition rate values for immune evasion $\rho$ of the SBM resulting from fitting the model to experimental data of either *C. albicans* or *S. aureus* infection in blood samples from HLM patient.

Despite the quantitative increase in leukocyte numbers and their functional changes, a fraction of *C.* *albicans* and *S.* *aureus* cells was not phagocytosed by neutrophils or monocytes even after 4 h of infection, and remained extracellular (Fig. 7). Comparing between different sample time points, we observed for both pathogens that the percentages of extracellular pathogens were decreased after surgery and the slope of the reaction curve was increased during the initial phase. Specifically, 60 min after inoculation $34.5 \pm 17.5\%$ of total *C.* *albicans* cells were not associated with immune cells in pre-operative blood (Fig. 7a). However, in blood samples collected immediately (post-operative) and one day after surgery, only $9.9\pm 7.8\%$ and $10.1\pm 3.7\%$ *C.* *albicans* cells, respectively, were not associated with immune cells at 60 min post infection (Fig. 7b,c)*.* Although these results indicated that elimination of pathogens from the extracellular space was more efficient after surgery, pathogen-specific differences were present in the blood under the three tested conditions. In every case, the percentage of extracellular cells was higher for *C.* *albicans* than for *S. aureus* following 240 min of infection (Fig. 7a–c). Our model predicted that at 240 min all extracellular cells of both pathogens would have undergone immune escape (${P}_{IE}$). Interestingly, despite increased neutrophil numbers and neutrophil phagocytosis rates in blood collected after surgery, estimated transition rates for acquiring immune escape ($\rho$) were twofold higher for *C.* *albicans* in post-operative compared to pre-operative samples, which was in contrast to *S.* *aureus*, which had almost equal $\rho$ values for the different sample time points (Fig. 7d, Table 1). In fact, this resulted in a changed ratio of $\rho$ between *S. aureus* and *C. albicans* infection in post-operative blood. While $\rho$ values were similar for *C.* *albicans* and *S.* *aureus* in pre-operative blood (${\rho }^{S.a.}/{\rho }^{C.a.} = 0.96$), larger differences could be detected in blood samples taken after surgery (post-operative: ${\rho }^{S.a.}/{\rho }^{C.a.} = 0.48$, post-operative + 1d: ${\rho }^{S.a.}/{\rho }^{C.a.} = 0.5$).

## Discussion

In this study, we applied a systems biology approach, where experimental whole-blood infection assays were combined with virtual infection modeling to investigate the effect of immune activation on the immune defense against pathogens in whole blood. In order to analyze the immune response of patients that suffer from imbalanced immune homeostasis, we used blood samples of patients who underwent cardiac surgery with extracorporeal circulation. The surgical intervention and the cardiopulmonary bypass are known to trigger a systemic inflammatory response. Although this transitory inflammation is not usually related to infection or sepsis, it can contribute to post-operative complications such as organ dysfunction or bleeding^23^. Pathophysiologically, a multitude of triggers that include surgical trauma, ischemia and reperfusion injury as well as endotoxinemia and glucanemia results in an acute phase reaction largely but not exclusively mediated by activation of NF-κB^20,24^. In particular, the inflammatory stimulus causes a surplus of the complement protein C5a, which serves as an important chemoattractant for several innate immune cells, including neutrophils and monocytes^25,26^. C5a molecules activate neutrophils and induce neutrophil degranulation and superoxide generation^26–31^. Due to this pro-inflammatory stimulus, patients show leukocytosis after cardiac surgery, including mobilization of neutrophils from the bone marrow. Furthermore, their monocytes express reduced levels of HLA-DR and increased CD62L^32^. Thus, transient hyperinflammation can be studied well in elective cardiac bypass surgery patients.

In accordance with this, we observed leukocyte recruitment after surgery in our study. In particular, the number of neutrophils increased more than twofold due to the recruitment of CD10^neg^ immature neutrophils. In addition, monocytes were increased at day one, but already showed an immature phenotype in blood taken directly after surgery. Despite the higher abundance of immature neutrophils and monocytes, pathogen association with immune cells occured faster and to a larger extent in post-operative compared to pre-operative blood samples. By calibrating the state-based virtual infection model to the association kinetics of samples taken before and after surgery, we found that this was not solely due to increased immune cell numbers. Rather, phagocytosis rates of neutrophils and monocytes increased by twofold after surgery, indicating an enhanced activation state. These quantitative results showing the increase in immune cell activity could not be detected by selectively quantifying surface activation marker expression, such as the neutrophil degranulation marker CD66b. Despite the high abundance of immature neutrophils recruited in response to transient hyperinflammation, these cells showed the same maximum of CD66b and CD69 up-regulation and decrease in surface CD16 expression upon fungal phagocytosis as mature neutrophils in pre-operative blood. Immature neutrophils contain three types of granules and can produce reactive oxygen species^33,34^, indicating that these cells are a functional subset that is released under certain conditions. Studies by Leliefeld et al*.* and van Grinsven et al. using experimentally-induced acute inflammation in healthy subjects by intravenous administration of bacterial LPS have demonstrated that the different neutrophil subsets not only differ in phenotype but also in function^35,36^. Immature neutrophils were shown to have even higher antibacterial capacity in vitro than mature neutrophils, and exhibit efficient migration, which led to the hypothesis that immature neutrophils are not released as bystanders but rather as cells that are more efficient in pathogen killing, in line with the observed increased immune cell activity in our study. Other previous studies have reported an increase in immune cell activation for monocytes and neutrophils after surgery, by evaluating the expression of immune cell specific activation markers and the cytokine profile^37–39^. However, they did not investigate quantitative changes in the immune cell functionality during the response to different pathogens serving as stimuli. The observed functional activation also mitigates pathogen-specific patterns of immune activation. Prior to the inflammatory insult and as observed in samples from healthy control donors, *S. aureus* infection induced a faster immune response than *C.* *albicans* infection. In contrast, after surgery, the inter-pathogen differences were substantially reduced.

Additionally, we analyzed the concentration of pro- and anti-inflammatory cytokines in blood samples of HLM patients before and after surgery. Interestingly, the pro-inflammatory cytokines IL-1β and TNF-α were not present in higher levels after surgery, whereas an increase was observed for others, such as IL-6. In addition, the anti-inflammatory cytokine IL-10 was also significantly increased at the end of surgery, indicating the activation of anti-inflammatory pathways. These findings are in line with several studies reporting the surgery-induced release of both pro- and anti-inflammatory mediators^27,40–43^. During ex vivo infection, the release of monocytic cytokines and the up-regulation of early activation antigen CD69 on monocytes were markedly lower in post-operative blood. Together with the immature phenotype of blood monocytes, this points towards a regulatory effect of increased IL-10 levels in blood taken directly after surgery. Among the mediators released during surgery, IL-10 has already been shown to contribute to the down-regulation of HLA-DR on CD14^+^ cells^44^. IL-10 levels decreased after one day, which consequently resulted in the partial recovery of HLA-DR on monocytes and secretion of cytokines IL-1β, IL-6 and TNF-α during both *C. albicans* and *S. aureus* infection.

Similar to results of our previous studies on the immune response in whole-blood samples upon *C. albicans* infection^16^, we observed a specific population of pathogens that could not be phagocytosed by the immune cells, but were still present in the extracellular space at four hours post-infection. Therefore, these pathogens must escape the immune defense. Although the evasive pathogens could be detected in stimulated blood samples at each sample time point before and after surgery, we found that the amount of evasive *C. albicans* cells and *S. aureus* cells decreased after surgery, while the rate for this process increased for *C. albicans* infection and remained almost unchanged for *S. aureus* infection. In a previous study, we investigated the mechanism of *C. albicans* immune evasion in whole blood by testing potential evasion mechanisms using mathematical modeling. By simulating the infection in whole blood under neutropenic conditions, we could suggest future experimental measurements that would most likely enable us to accept or reject one of the two tested mechanisms, i.e. spontaneous evasion or neutrophil-mediated immune evasion^45^. Since we observed larger evasion rates after surgery, where the immune cells are more active in terms of phagocytosis, our study provides clues that the evasion of pathogens could be dependent on immune cell activity. Moreover, the change in the immune evasion rate after surgical insult is an additional characteristic property describing the changes of the immune response in whole-blood samples of patients who experienced an inflammatory stimulus.

Taken together, we show that a combination of experimental assays in ex vivo blood samples and biomathematical monitoring is a feasible approach for immune profiling in patients. Clearly, at this stage this cannot be used for diagnostic purposes and requires both further optimization and standardization. However, using this approach in its current state, we can already reveal important pathophysiological properties governing virulence of microorganisms, as in the case of immune escape of *C. albicans*.

## Methods

### Experimental methods

#### Patients and ethics statement

Human peripheral blood was collected from healthy volunteers and heart lung machine (HLM) patients with written informed consent. This study was conducted in accordance with the Declaration of Helsinki and all protocols were approved by the Ethics Committee of the University Hospital Jena (permit number: 4643-12/15).

Patients included in this study received standardized minimally invasive cardiac surgery for mitral valve insufficiency with a heart lung machine and same anesthesia regimen. Blood was taken from inserted catheters before cardiac surgery (pre-operative), immediately after surgery (post-operative) and one day after admission to the intensive care unit (post-operative + 1d).

#### Strains and culture

*Candida albicans* (SC5314) was grown overnight in YPD medium (2% D-glucose, 1% peptone, 0.5% yeast extract, in water) at 30 °C to stationary phase. Fungal cells were reseeded in YPD medium, grown for 3 h at 30 °C, stained with CellTracker™ Green (Invitrogen) for 1 h, and harvested in PBS. Subsequently, cells were inactivated with 0.1% thimerosal at 37 °C for 1 h and then rinsed extensively.

*Staphylococcus aureus* (ATCC25923) was cultivated overnight in lysogeny broth (LB) medium (10 g/l tryptone, 5 g/l yeast extract, 10 g/l sodium chloride, pH 7) at 37 °C. Bacterial cells were reseeded in LB medium and grown at 37 °C to reach the exponential growth phase (OD_600_ of 0.6–0.7) followed by staining with CellTracker™ Green for 30 min and inactivation with 50% ethanol for 4 h at 37 °C. Both pathogen stocks were stored at − 20 °C until use.

#### Ex vivo whole-blood infection assay

Peripheral blood from healthy volunteers and from patients undergoing cardiac surgery with extracorporeal circulation was collected in S-monovettes® (Sarstedt) containing recombinant Hirudin as anti-coagulant. Differential blood cell counts were measured with an auto hematology analyzer (BC-5300, Mindray).

For whole-blood infection, PBS as mock-infected control (mock) or 1 × 10^6^ killed pathogens per ml whole blood were incubated for various time points (as indicated) at 37 °C on a rolling mixer (5 rpm). After incubation, the samples were immediately placed on ice. To collect plasma samples, whole-blood aliquots were centrifuged for 10 min at 10,000 × *g* and 4 °C, and the resulting plasma was stored at − 20 °C until further analysis.

#### Flow cytometry

Differential staining and flow cytometry was applied to identify distinct immune cell populations and to measure activation of immune cells and their association to pathogens. For surface antigen staining on the different immune cells, whole blood was stained with mouse anti-human CD3 (clone SK7, T cells), CD19 (clone HIB19, B cells), CD56 (clone MEM-188, NK cells), CD66b (clone G10F5, neutrophils), obtained from BioLegend, and CD69 (clone L78, early activation antigen, BD Biosciences). Monocytes were labelled with anti-human CD14 antibody (clone REA599, Miltenyi Biotec). Immature phenotype of neutrophils was assessed by surface CD10, CD16 and CD62L expression using mouse anti-human CD10 (clone HI10a, BioLegend), CD16 (clone 3G8, BioLegend) and CD62L (clone REA615, Miltenyi Biotec) antibodies. Surgery-induced changes on monocyte surface antigen expression were analysed for HLA-DR (anti-human HLA-DR, clone REA805, Miltenyi Biotec) and CD62L. Red blood cells were lysed with BD FACS Lysing solution followed by washing and harvesting cells in BD CellWASH solution. Acquisition was performed with the BD FACSCanto II flow cytometer and data was processed with FlowJo 7.6.4 software.

The strategy used to evaluate the association of microorganisms to immune cells in human blood was shown for GFP-expressing *C.* *albicans* in Hünniger et al., 2014 and used in the same way for CellTracker™ Green-labeled *C.* *albicans* and *S.* *aureus* in this study^16^.

#### Quantification of cytokines

The secretion of cytokines was assessed in plasma samples using Luminex technology (ProcartaPlex™ Multiplex Immunoassay, Thermo Fisher Scientific). The analyses were performed according to the instructions from the manufacturer.

#### Statistical analyses

Data are presented as arithmetic means ± standard deviation (SD). Statistical analysis was performed by applying the following steps. First, the Shapiro–Wilk test was applied to test whether the underlying data is normally distributed. For normally distributed data the unpaired *t*-test was used to test for significant differences, since the respective data was unpaired. If the data was not normally distributed, either the Wicoxon signed-rank test was applied to test paired samples, or the Mann–Whitney U test was applied to test unpaired samples for significant differences. Afterwards, a multiple comparison correction (Bonferroni’s correction) was performed if comparisons were made between several data sets. The corrected *P*-value is given by $P^{\prime}.$ Significance is shown as *P < 0.05, **P < 0.01, ***P < 0.001.

### Biomathematical modeling

#### State-based virtual infection modelling

The state-based model (SBM) is derived from our previous models of whole-blood infection^16–18^ and simulates the immune defence in whole blood that was infected with killed pathogens. As depicted in Supplementary Fig. S1a, the SBM contains several states for distinct cell populations in the system. Killed pathogens in extracellular space are represented by the state ${P}_{KE}$ and pathogens that acquired immune escape are described by the state ${P}_{IE}$. Furthermore, the model contains the states ${N}_{j}$ and ${M}_{j}$ that represent neutrophils and monocytes, respectively, where the index $j$ denotes the number of phagocytosed pathogens. In order to compare the dynamics of the model with experimental data, we defined combined units that are specific combinations of measurable model states. Pathogens in extracellular space are represented by the combined unit ${P}_{E}$:

$${P}_{E}={P}_{IE}+{P}_{KE}.$$

The combined units ${P}_{N}$ and ${P}_{M}$ refer to pathogens that have been phagocytosed by either neutrophils or monocytes:

$${P}_{N}=\sum_{j=1}^{j=n}{N}_{j}\times j,\mathrm{ and}$$

$${P}_{M}=\sum_{j=1}^{j=n}{M}_{j}\times j.$$

The transitions between model states represent biological processes during whole-blood infection (see the connections between states in Supplementary Fig. S1a). In the SBM, we defined three transition rates characterising specific transitions. The rate $${\phi }_{N}$$ refers to the phagocytosis of killed pathogens ($${P}_{KE}$$) by neutrophils with $$j$$ phagocytosed pathogens ($${N}_{j})$$ and characterises the state transition

$${N}_{j}+ {P}_{KE}\to {N}_{j+1}.$$

The phagocytosis of killed pathogens ($${P}_{KE}$$) by monocytes with $$j$$ phagocytosed pathogens ($${M}_{j})$$ is characterised by the rate $${\phi }_{M}$$ and is described by

$${M}_{j}+ {P}_{KE}\to {M}_{j+1}.$$

The immune escape of killed pathogens (${P}_{KE}$) is quantified by the rate $\rho$ for state transition

$${P}_{KE}\to {P}_{IE}.$$

The transition rates enables us to quantify the dynamics of the SBM, where a transition from any state $S$ to state ${S}^{^{\prime}}$ within the time step $\Delta t$ will be performed with probability ${P}_{S\to {S}^{^{\prime}}}$: ${P}_{S\to S{^{\prime}}}= {r}_{S\to S{^{\prime}}}\times\Delta t$. The SBM dynamics was calculated by applying the random selection method^46^ and the flow chart of this simulation algorithm is depicted in Supplementary Fig. S1b.

#### Agent-based virtual infection modelling

In order to investigate also spatial aspects of host–pathogen interactions we applied a previously developed agent-based model (ABM)^47,48^ that was adjusted to the context of whole-blood infection assays^17,18^. This spatial counterpart of the SBM simulates the different immune cell types of neutrophils and monocytes as well as the killed pathogens as distinct spherical objects, i.e. the agents (Supplementary Fig. S2a). These agents can migrate and interact within an environment in a rule-based fashion, where the environment is a continuous three-dimensional representation of a section of $0.5 \mu \mathrm{l}$ of the whole-blood infection assay in which cells perform random walk migration due to the high density of erythrocytes^17^. Modeling the active migration of neutrophils and monocytes by a diffusion process with coefficients ${D}_{N}$ and ${D}_{M}$, respectively, the passive migration of pathogens is estimated to have a small diffusion coefficient of ${D}_{P}\approx 1 \mu {\mathrm{m}}^{2}/\mathrm{min}$ ^17^. Based on the random selection method^46^, each stochastic simulation was repeated 30 times to obtain significant mean and standard deviation of simulation dynamics (Supplementary Fig. S2b). The migration parameters of immune cells were estimated from a minimization of least-squares error (LSE) between experiment and simulation using the method of *adaptive regular grid search*^49^.

#### Parameter estimation

In order to estimate the a priori unknown values of transition rates, the state-based model (SBM) is fitted to experimental data of the whole-blood infection assays by applying the algorithm of *simulated annealing*^50^ based on the *Metropolis Monte Carlo* scheme^51^. For a detailed description of the parameter estimation in the SBM we refer to our previous work^17,18,29,45^ and to the flowchart of this parameter fitting algorithm in Supplementary Fig. S3.

To calibrate the model parameters of the agent-based model (ABM) to the experimental data, we previously developed a bottom-up approach that combines the SBM and ABM such that estimated rates from the SBM can be used in the ABM by a self-consistent conversion of these parameters^17^. Therefore, the parameter search space reduces to the migration parameters of the immune cell types ${D}_{N}$ and ${D}_{M}$. For a detailed description of the parameter estimation in the ABM we refer to our previous work^17,18,29,45^ and the flowchart of this algorithm in Supplementary Fig. S4.

## Supplementary Information

Supplementary Information.
