# Supplementary material for: Ex vivo immune profiling in patient blood enables quantification of innate immune effector functions
Source: Sci Rep. 2021 Jun 8;11:12039. doi: 10.1038/s41598-021-91362-5 (PMC8187451; doi:10.1038/s41598-021-91362-5)
Supplement: Supplementary file 1 — Supplementary Information. [file 41598_2021_91362_MOESM1_ESM.pdf]

Ex vivo Immune Profiling in Patient Blood Enables Quantification of Innate Immune Effector Functions

Teresa Lehnert[1,2,#], Ines Leonhardt[2,3,#], Sandra Timme[1], Daniel Thomas-Rüddel[2,4], Frank Bloos[2,4], Christoph Sponholz[4], Oliver Kurzai[2,3,5], Marc Thilo Figge[1,2,6,##,*], Kerstin Hünniger[3,5,##,*]

[1] Research Group Applied Systems Biology, Leibniz Institute for Natural Product Research and Infection Biology - Hans Knöll Institute, Jena, Germany.

[2] Center for Sepsis Control and Care (CSCC), Jena University Hospital, Jena, Germany.

[3] Research Group Fungal Septomics, Leibniz Institute for Natural Product Research and Infection Biology - Hans Knöll Institute, Jena, Germany.

[4] Department of Anesthesiology and Intensive Care Medicine, Jena University Hospital, Jena

[5] Institute for Hygiene and Microbiology, University of Würzburg, Würzburg, Germany.

[6] Institute of Microbiology, Faculty of Biological Sciences, Friedrich Schiller University Jena, Jena, Germany.

[#] Teresa Lehnert and Ines Leonhardt are joint first authors and contributed equally to this work. The order was determined alphabetically.

[##] Marc Thilo Figge and Kerstin Hünniger are joint senior authors and contributed equally to this work. The order was determined alphabetically.

[*] Address correspondence to:
Marc Thilo Figge
e-mail address: thilo.figge@leibniz-hki.de
phone number: 0049 3641 5321416
fax number: 0049 3641 5322416

Kerstin Hünniger
e-mail address: kerstin.huenniger@leibniz-hki.de
phone number: 0049 3641 5321361
fax number: 0049 3641 9396502

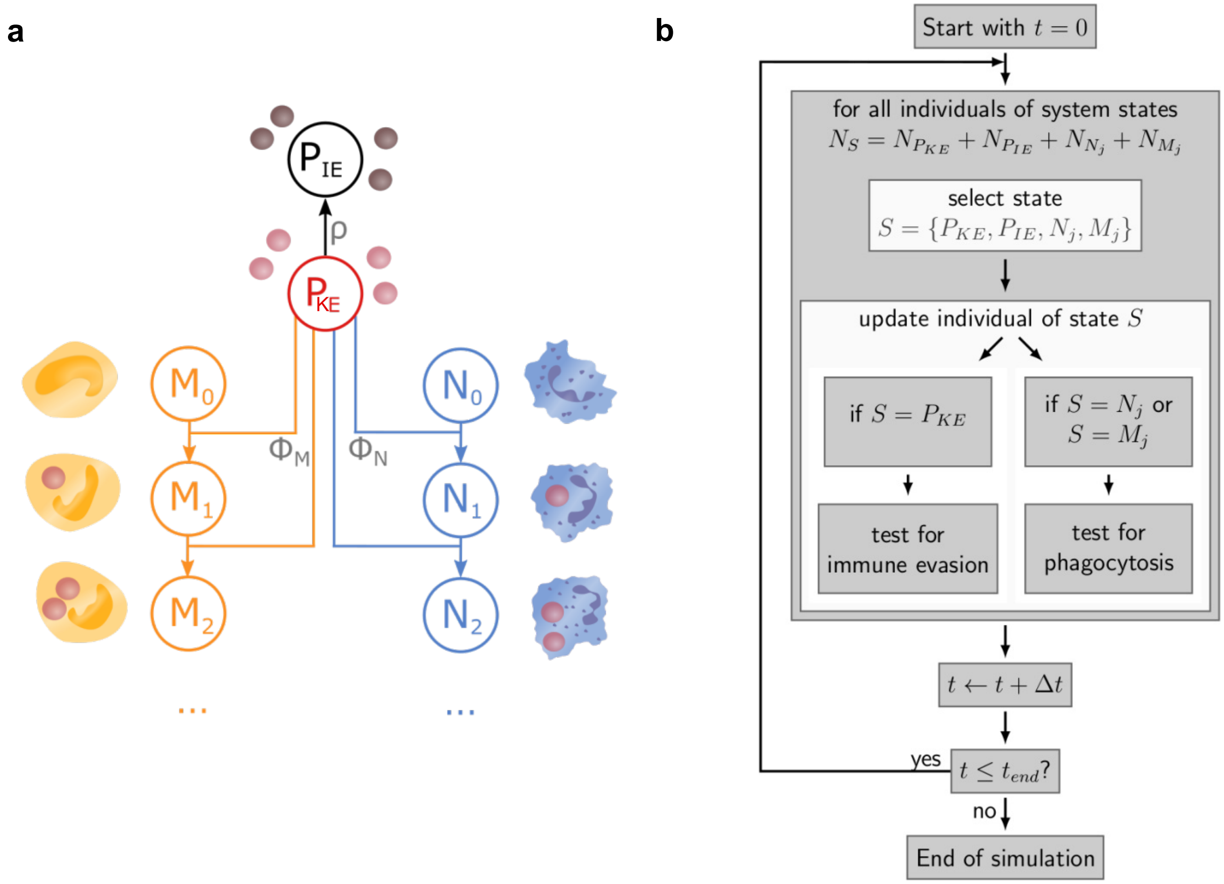

Supplementary Figure S1. Schematic depiction of the state-based simulation algorithm (a) and the state-based virtual infection model for whole-blood infection (b). (a) The model is composed of different states that represent several cell populations during whole-blood infection (colored circles). The model contains states for killed pathogens in extracellular space (P_{KE}), immune evasive pathogens (P_{IE}) as well as two types of immune cells, neutrophils (N_j) and monocytes (M_j) with *j* phagocytosed pathogens. Connections between the states indicate possible state transitions that are characterized by transition rates (grey colored Greek letters): ρ for the acquisition of immune escape as well as ϕ_N and ϕ_M for phagocytosis by neutrophils and monocytes. The figure is adapted from Hünniger *et al.,* 2014. (b) For each simulation time *t*, an individual is randomly selected out of the number of individuals N_S. The current state of the individual can be left in dependence on the respective rate of the transition. After testing each individual for possible state transition, the simulation time is increased by the simulation time step Δt and the simulation algorithm ends if the simulation time t_{end} is reached. The figure is adapted from Lehnert *et al.*, 2015.

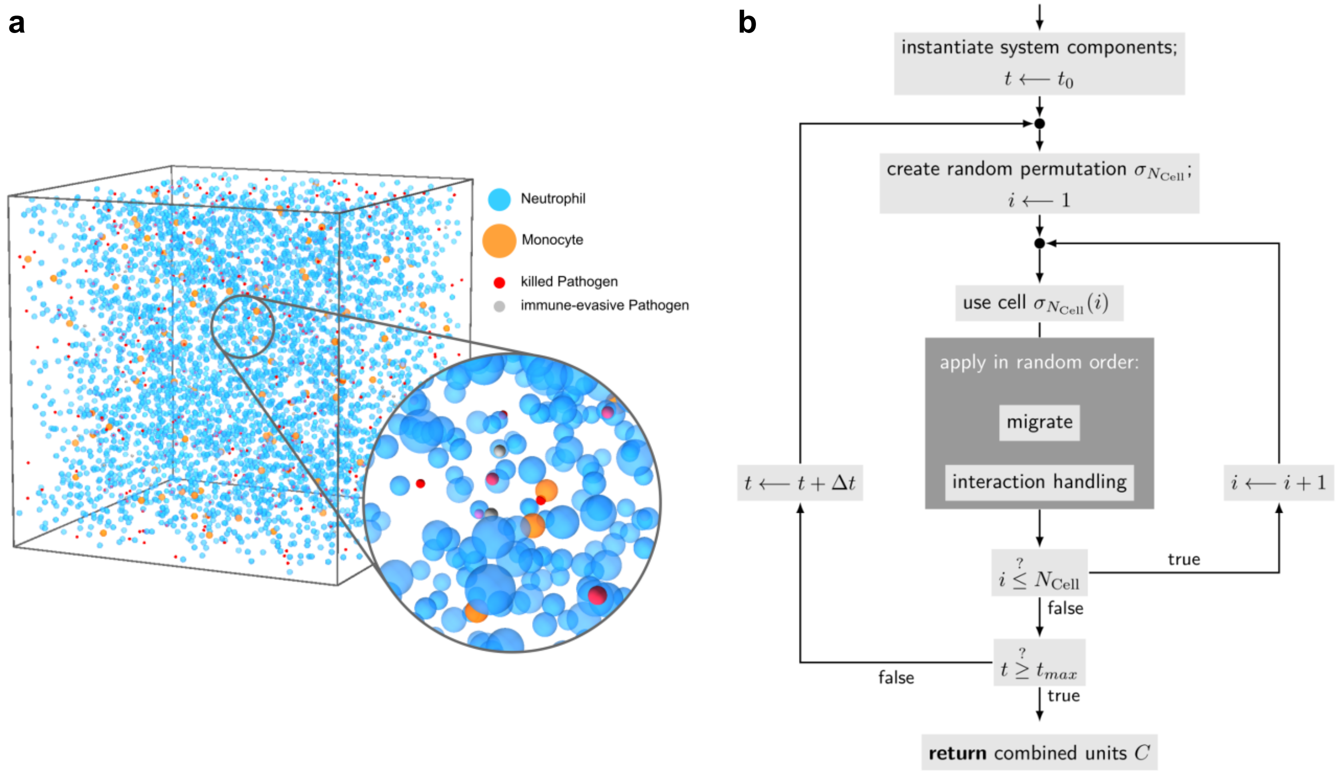

Supplementary Figure S2. Schematic depiction of the agent-based virtual infection model for whole-blood infection (a) and its simulation algorithm (b). (a) Both immune cell types – monocytes (orange) and neutrophils (blue) – as well as the pathogenic cells (red: killed pathogens, grey: immune evasive killed pathogens) are modeled as spherical objects within a continuous three-dimensional environment representing 0.5 µl of blood. The figure is adapted from Lehnert *et al.,* 2015. (b) First, in the ABM all system components are instantiated. Afterwards, the interaction of monocytes and neutrophils with the pathogen is simulated for a time t_{max}. During each discrete time step t for all cells migration and interaction with other cells in the environment is simulated in random order. The figure is adapted from Lehnert *et al.*, 2015.

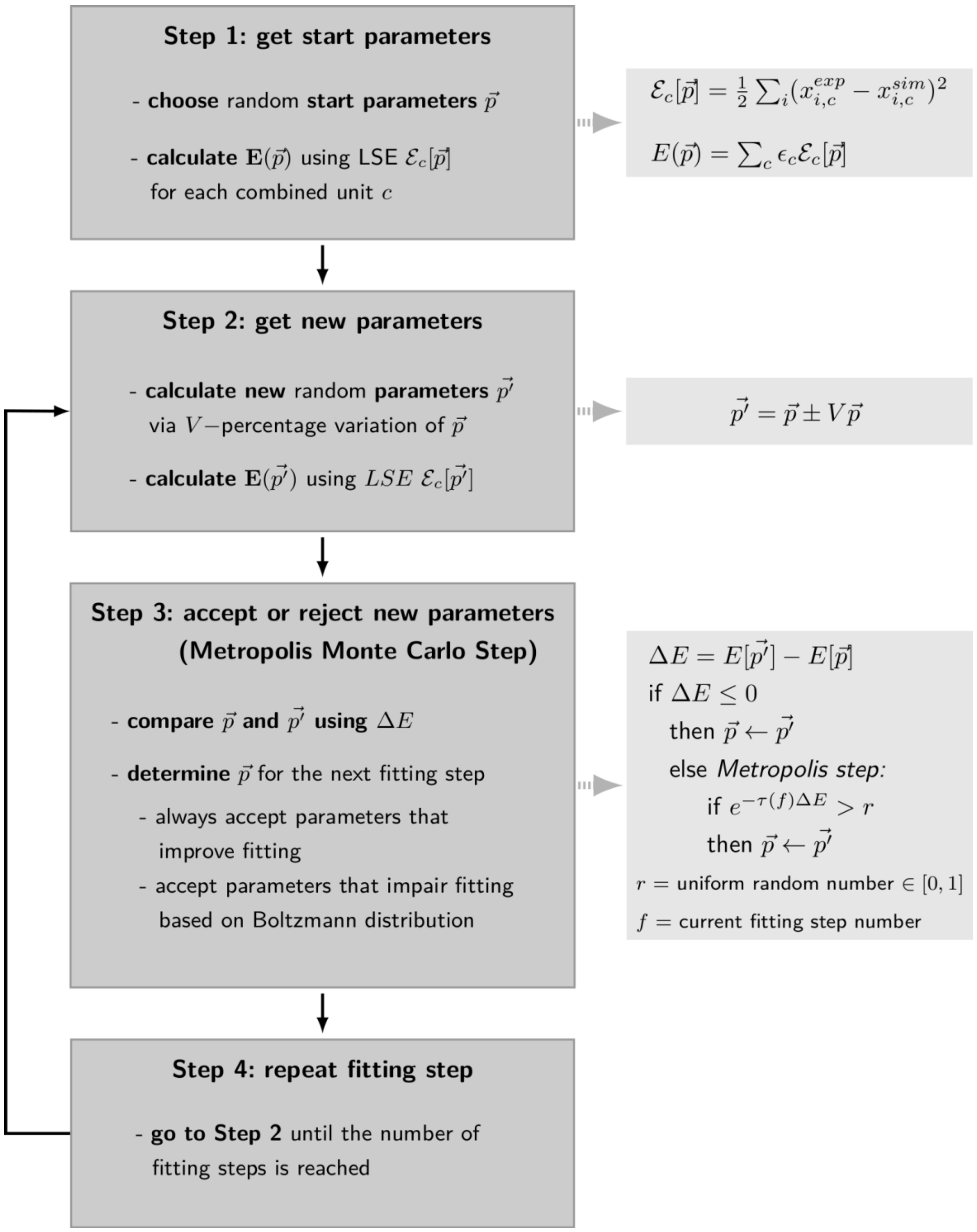

Supplementary Figure S3. Flow chart of the SBM parameter estimation algorithm. Calibration of the SBM to the experimental data is realized by the Metropolis Monte-Carlo algorithm with simulated annealing. The algorithm is explained in detail in the Methods section *Parameter estimation*. The flowchart is adapted from Lehnert *et al.* 2015.

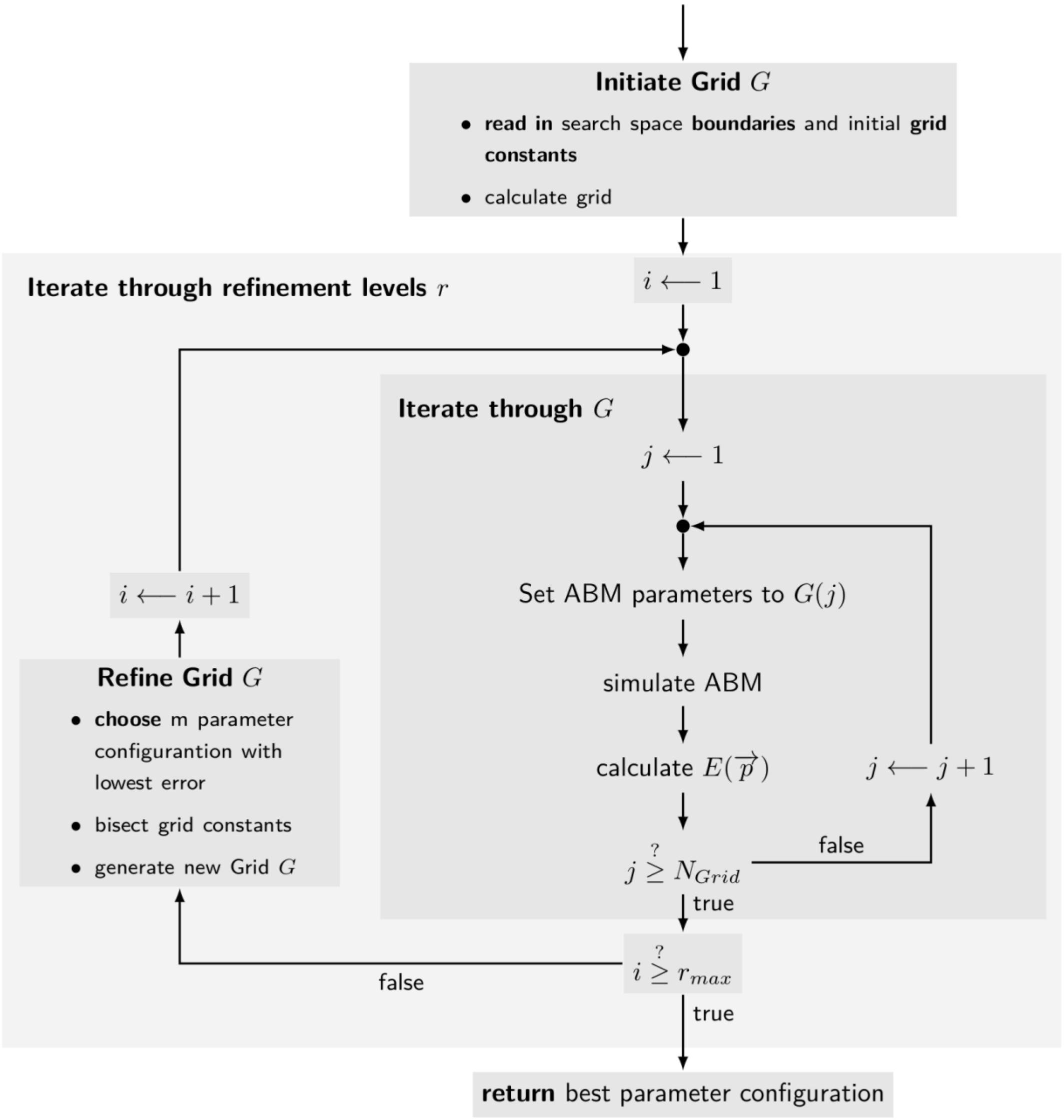

Supplementary Figure S4. Flow chart of the ABM parameter estimation algorithm. For calibration of the ABM to the experimental data the local method of *adaptive regular grid search* is applied. First, a grid within the parameter space with predefined boundaries is initiated with a certain grid constant. Afterwards, the algorithm iterates through the grid and the model is simulated with the current parameter $\vec{p}$. The deviation of the simulation outcome and the experimental data is evaluated using the weighted least squares error. Furthermore, around the best parameters $\vec{p}$, *i.e.* the parameters with a low $E(\vec{p})$, the parameter space is screened with a more fine-grained grid by bisecting the current grid constant. The flowchart is adapted from Lehnert *et al.* 2015.

	pre-op.	post-op.	post-op.+1d	
**Donor 1**				
WBC (x 10^9 / L)	3.43	7.06	9.77	
Neutrophils (x 10^9 / L)	1.77	5.74	7.36	
Lymphocytes (x 10^9 / L)	1.37	1.06	1.87	
Monocytes (x 10^9 / L)	0.17	0.19	0.41	
RBC (x 10^{12} / L)	4.47	4.11	4.58	
HCT	0.38	0.36	0.40	
CPB time (min)				99
Cumulative fluid balance on the day of surgery (ml)				3,000-3,500
Transfusions				no
**Donor 2**				
WBC (x 10^9 / L)	5.46	10.54	14.04	
Neutrophils (x 10^9 / L)	3.25	8.85	12.53	
Lymphocytes (x 10^9 / L)	1.55	1.42	0.85	
Monocytes (x 10^9 / L)	0.40	0.13	0.64	
RBC (x 10^{12} / L)	3.54	3.34	3.39	
HCT	0.32	0.31	0.30	
CPB time (min)				122
Cumulative fluid balance on the day of surgery (ml)				0-500
Transfusions				no
**Donor 3**				
WBC (x 10^9 / L)	4.33	7.93	10.07	
Neutrophils (x 10^9 / L)	2.41	6.13	8.59	
Lymphocytes (x 10^9 / L)	1.55	1.42	0.76	
Monocytes (x 10^9 / L)	0.16	0.17	0.69	
RBC (x 10^{12} / L)	4.35	3.53	3.16	
HCT	0.43	0.36	0.31	
CPB time (min)				112
Cumulative fluid balance on the day of surgery (ml)				2,500-3,000
Transfusions				no

	pre-op.	post-op.	post-op.+1d	
**Donor 4**				
WBC (x 10^9 / L)	3.91	9.99	12.65	
Neutrophils (x 10^9 / L)	2.54	8.12	10.90	
Lymphocytes (x 10^9 / L)	1.16	1.58	1.13	
Monocytes (x 10^9 / L)	0.06	0.16	0.46	
RBC (x 10^{12} / L)	4.01	2.74	3.24	
HCT	0.37	0.26	0.30	
CPB time (min)				183
Cumulative fluid balance on the day of surgery (ml)				3,000-3,500
Transfusions				1xRCC, 1xPLC
**Donor 5**				
WBC (x 10^9 / L)	3.96	12.62	7.09	
Neutrophils (x 10^9 / L)	1.97	11.65	5.81	
Lymphocytes (x 10^9 / L)	1.66	0.62	1.01	
Monocytes (x 10^9 / L)	0.19	0.29	0.22	
RBC (x 10^{12} / L)	5.15	3.83	3.77	
HCT	0.47	0.36	0.34	
CPB time (min)				107
Cumulative fluid balance on the day of surgery (ml)				2,000-2,500
Transfusions				no
**Donor 6**				
WBC (x 10^9 / L)	7.12	11.06	11.69	
Neutrophils (x 10^9 / L)	4.34	9.53	9.61	
Lymphocytes (x 10^9 / L)	2.10	1.09	1.14	
Monocytes (x 10^9 / L)	0.49	0.33	0.79	
RBC (x 10^{12} / L)	3.94	3.19	2.61	
HCT	0.35	0.29	0.23	
CPB time (min)				220
Cumulative fluid balance on the day of surgery (ml)				7,500-8,000
Transfusions				3xRCC, 2xPLC, 4xFFP

Supplementary Table S1. Blood samples from HLM patients taken before cardiac surgery (pre-op.), immediately after surgery (post-op.) and one day after admission to intensive care (post-op.+1d) were analyzed for white blood cell (WBC), neutrophil, lymphocyte, monocyte and red blood cell (RBC) counts as well as hematocrit (HCT) using an automated hematology analyzer. Cardiopulmonary bypass (CPB) time, cumulative fluid balance on the day of surgery and given blood transfusions (red cell concentrate, RCC; platelet concentrate, PLC; fresh frozen plasma, FFP) for each patient are provided.
